# Comprehensive pan-cancer analysis of inflammatory age-clock-related genes as prognostic and immunity markers based on multi-omics data

**DOI:** 10.1038/s41598-024-61381-z

**Published:** 2024-05-07

**Authors:** Bo Yan, Pan Liao, Shan Liu, Ping Lei

**Affiliations:** 1 Haihe Laboratory of Cell Ecosystem, Department of Geriatrics, Tianjin Medical University General Hospital, 154 Anshan Road, Heping District, Tianjin, 300052 China; 2 Tianjin Geriatrics Institute, Tianjin Medical University General Hospital, 154 Anshan Road, Heping District, Tianjin, 300052 China; 3 The School of Medicine, Nankai University, 94 Weijin Road, Tianjin, 300071 China

**Keywords:** Pan-cancer, Inflammatory age, Epigenetic, Genomics, Immunogenomic, Risk model, Cancer, Computational biology and bioinformatics, Genetics, Immunology

## Abstract

Inflammatory age (iAge) is a vital concept for understanding the intricate interplay between chronic inflammation and aging in the context of cancer. However, the importance of iAge-clock-related genes (iAge-CRGs) across cancers remains unexplored. This study aimed to explore the mechanisms and applications of these genes across diverse cancer types. We analyzed profiling data from over 10,000 individuals, covering 33 cancer types, 750 small molecule drugs, and 24 immune cell types. We focused on DCBLD2’s function at the single-cell level and computed an iAge-CRG score using GSVA. This score was correlated with cancer pathways, immune infiltration, and survival. A signature was then derived using univariate Cox and LASSO regression, followed by ROC curve analysis, nomogram construction, decision curve analysis, and immunocytochemistry. Our comprehensive analysis revealed epigenetic, genomic, and immunogenomic alterations in iAge-CRGs, especially DCBLD2, leading to abnormal expression. Aberrant DCBLD2 expression strongly correlated with cancer-associated fibroblast infiltration and prognosis in multiple cancers. Based on GSVA results, we developed a risk model using five iAge-CRGs, which proved to be an independent prognostic index for uveal melanoma (UVM) patients. We also systematically evaluated the correlation between the iAge-related signature risk score and immune cell infiltration. iAge-CRGs, particularly DCBLD2, emerge as potential targets for enhancing immunotherapy outcomes. The strong correlation between abnormal DCBLD2 expression, cancer-associated fibroblast infiltration, and patient survival across various cancers underscores their significance. Our five-gene risk signature offers an independent prognostic tool for UVM patients, highlighting the crucial role of these genes in suppressing the immune response in UVM.Kindly check and confirm whether the corresponding affiliation is correctly identified.I identified the affiliation is correctly.thank you.Per style, a structured abstract is not allowed so we have changed the structured abstract to an unstructured abstract. Please check and confirm.I confirm the abstract is correctly ,thank you.

## Introduction

Cancer is regarded as an aging-related disease and is the leading cause of mortality in elderly people^1^. Tumorigenesis is an evolutionary process involving sequential steps, such as genetic changes, abnormal immune functions, and chronic inflammation^2–5^. Mounting evidence has shown a correlation between the senescent microenvironment, cancer progression, and metastasis^6–12^. Studies have demonstrated that aging could be a risk factor for the onset of glioblastoma multiforme (GBM) and could indicate its prognosis^13–15^.

While chronological age often serves as a conventional marker for aging, the relationship between health status and an individual’s ability to perform physical activity is complex and may not correlate perfectly with chronological age. In fact, individuals of the same age can exhibit vastly different physiological conditions, and individuals of different ages may share similar physiological characteristics. This complexity has led to the exploration of biological age as a more accurate predictor of age-related disorders. Aging, as a natural process, shares common features with cancer, such as the exhaustion of stem cells, low-grade inflammation, and damage due to proinflammatory factors^16–18^.

In 1863, Rudolf Virchow proposed that chronic inflammation could cause cancer^19^. Studies have since established a complex correlation between cancer and inflammation. Inflammation can be induced by various factors, including oncogenes, radiation, and senescence, in GBM, with inflammatory cells secreting cytokines that promote tumor growth, angiogenesis, and metastasis^20,21^. The significance of inflammation in cancer is well recognized, and anti-inflammatory drugs have shown promise in reducing the risk of certain cancers^22^. However, there are no standardized methods for monitoring inflammatory cells or reference values for inflammatory age (iAge) in cancer patients. To address the need to measure biological age and explore its potential implications in cancer, a research group estimated systemic iAge by constructing a metric known as the iAge clock^23^. Similar to the epigenetic and transcriptomic clocks, the iAge clock has the capacity to reflect multifaceted aging phenotypes and has promising clinical applications. However, its specific utility in the context of cancer has yet to be fully explored.

Given the intricate connections among aging, inflammation, and cancer, this study aimed to comprehensively analyze the genetic, immune, and clinical characteristics related to iAge-clock-related genes (iAge-CRGs) across 33 different cancer types. Our results revealed the impact of epigenetic alterations, genomic variations, and immunogenomic factors on the abnormal expression of these iAge-CRGs, with particular emphasis on DCBLD2. Additionally, we observed a significant correlation between aberrant DCBLD2 expression, the infiltration of immune cells (particularly cancer-associated fibroblasts), and overall survival in patients with various cancer types. Furthermore, based on the results of gene set expression analysis (GSVA), we constructed a risk model based on the five iAge-CRGs, which emerged as an independent prognostic index for uveal melanoma (UVM) patients. We systematically assessed the correlations of the risk score of the iAge-related signature with immune cell infiltration. Our findings elucidated the regulatory mechanisms of iAge-CRGs within the tumor microenvironment and suggest that these genes may serve as functional prognostic biomarkers, offering insights into the clinical outcomes of UVM patients and potential targets for increasing the efficacy of immunotherapy.

## Materials and methods

### Types of tumors and datasets

Changes in the genome and immune microenvironment are vital for regulating the development and progression of tumors and for the evaluation of disease diagnosis, disease outcomes, and treatment effectiveness in individual patients. In the era of abundant biological information, the expression of individual genes may be overshadowed by significant background interference. However, a comprehensive approach that combines the gene expression or gene set scores of multiple patients at various stages of tumorigenesis, drawn from diverse databases, can provide a comprehensive view of the underlying processes associated with cancer. Different omics data from related studies were used to expand the scope of the study^24,25^. Thus, integrating multi-omics data may identify the role of iAge-CRGs in different tumors more comprehensively. In this study, we analyzed changes in genes, such as gene expression changes, single nucleotide changes (SNVs), variations in gene copies (CNVs), and gene methylation status changes^26^. Additionally, we considered patients’ response to medications and the infiltration levels of 24 diverse immune cell types^27^.

To carry out this analysis, we obtained multi-omics data from several key sources: Information on immune cell infiltration (ICI, n = 10,995), gene expression (n = 10,995), clinical features (n = 11,160), disease staging (n = 9478), CNV (n = 11,495), and methylation profiles (450 k level 3) of patients were obtained from The Cancer Genome Atlas (TCGA; https://portal.gdc.cancer.gov/) and the UCSC Xena platform (http://xena.ucsc.edu/). From the Synapse database (https://www.synapse.org/ Synapse: syn7824274), we obtained information on single nucleotide variations (SNVs, n = 10,234), and from The Cancer Proteome Atlas (TCPA; https://tcpaportal.org/tcpa/index.html), we obtained information on reversed-phase protein arrays (RPPAs, n = 7876)^27^. We also obtained data from The Genomics of Drug Sensitivity in Cancer (GDSC; https://www.cancerrxgene.org/) and the Cancer Therapeutics Response Portal (CTRP; https://www.cancerrxgene.org/) databases to assess the correlation between iAge-CRG expression and patient sensitivity to drugs. The analysis covered a wide range of cancer types for a total of 33 different cancer types (Table S1), and the gene signatures of 24 immune cell types were assessed (Table S2)^27^. Through this comprehensive analysis, we identified a total of 38 iAge-CRGs (Fig. S1). Importantly, an independent investigation revealed a connection between these 38 iAge-CRGs and the likelihood of heart disease in adults^28^.

### Copy number variation analysis

The CNV Summary module was used to provide an overview of CNVs in selected cancers and reveals the genetic alterations associated with these diseases. To generate these summaries, we gathered CNV data from a comprehensive cohort of 11,495 patients from the TCGA. The GISTIC2.0 algorithm was utilized to identify regions within patient genes that were significantly amplified or deleted in our analysis. The GISTIC score serves as a key indicator of the CNV/gene relationship: − 2 denotes a deep deletion representing a profound loss or homozygous deletions, − 1 signifies a shallow deletion corresponding to a milder loss or heterozygous deletion, and 0 indicates a diploid state. Furthermore, a GISTIC score equal to or greater than one indicates a minimal increase, frequently linked to the procurement of a small number of extra duplicates involving extensive gains or heterozygous amplification. A score of two or higher signifies a high level of amplification, indicating the presence of more copies, potentially reaching homozygous amplification. The distribution of samples in four categories (GISTIC score: − 2, − 1, 1, 2) of CNV in specific cancer types was meticulously summarized.

To investigate the association between CNV and gene expression, we conducted a Spearman correlation analysis utilizing the approach outlined by Schlattl et al.^29^. By employing this method, we were able to evaluate the correlation between these two crucial elements. A comprehensive analysis was conducted by integrating data on RSEM-normalized gene expression data and CNV data from the TCGA by utilizing the TCGA barcode for data integration. To ensure the statistical rigor of our findings, p values adjusted according to the FDR.

### Single nucleotide variant analysis

The SNV summary module serves as a valuable resource for understanding SNVs within selected cancers. Our dataset (from the TCGA) included information on 10,234 patients with 33 distinct cancer types. Within this comprehensive dataset, our analysis focused on mutations that are particularly relevant to cancer research, namely, deleterious mutations. It is well known that deleterious mutations can disrupt normal gene function and contribute to the onset and development of cancer.

Conversely, nondeleterious mutations, which include silent mutations, intronic mutations, mutations in intergenic regions (IGRs), and those within 3ʹ and 5ʹ untranslated regions (UTRs), as well as 3ʹ and 5ʹ flanking regions, were excluded from our analysis. Nondeleterious mutations are typically considered less impactful in terms of their influence on gene function and cancer-related processes.

### Methylation analysis

The module for differential methylation acts as a valuable tool for comprehending the methylation conditions of cancer patients and their respective normal controls. We gathered Illumina HumanMethylation 450 k level 3 data from more than ten paired tumor and adjacent nontumor samples encompassing various cancer types (THCA, BLCA, ESCA, KIRP, LIHC, BRCA, HNSC, PRAD, KICH, LUSC, KIRC, STAD, LUAD, and COAD) obtained from the TCGA to compile this dataset. Each gene contains numerous methylation sites, and multiple tags are used to store data regarding the level of methylation at each site. A correlation analysis was conducted to identify methylation sites that exhibited a negative correlation with gene expression. T tests were used to estimate p values, which were then adjusted using FDR. The methylation and expression module were utilized for Spearman correlation analysis to establish the relationship between methylation levels and gene expression.

### Differential mRNA expression analysis

Differential mRNA expression analysis was performed as a critical step in our exploration of cancer-related gene expression patterns. The RNA-Seq (n = 10,995) and clinical characteristic (n = 11,160) data of patients were obtained from TCGA datasets. To conduct differential expression analysis, we incorporated RSEM gene expression data that were normalized and batch-corrected. These data included data on 13 paired tumor and normal samples from various cancer types, such as PRAD, KIRP, BLCA, THCA, HNSC, LIHC, LUAD, BRCA, ESCA, KICH, STAD, KIRC, LUSC, and COAD. The fold change (FC) in expression was calculated as follows: FC = mean (tumor)/mean (normal).

### Pathway activity analysis

To evaluate the differences in the enrichment of pathways between samples types, we assessed gene expression data and pathway scores. Samples were considered to exhibit activation or inhibition of pathways according to the median pathway scores. We used RPPA data from the TCGA to determine the activity scores of 10 pathways associated with cancer in 7876 patients across 32 various types of cancer^27^. We examined signaling pathways associated with apoptosis, the cell cycle, RTK, PI3K/AKT, TSC/mTOR, RAS/MAPK, ER and AR, epithelial-to-mesenchymal transition (EMT), and the DNA damage response.

Afterward, we assessed the comparative protein expression of each sample by utilizing median-centered RPPA-RBN data, which were subsequently normalized using standard deviation calculations. The pathway score was calculated by summing the protein expression of positive regulatory components in a specific pathway and subtracting the negative regulatory components using the method outlined in a previous study^30^.

Based on the median gene expression, we classified the patients into two groups: the low-gene expression group (LRG) and the high-gene expression group (HRG). We assessed the difference in the pathway activity score (PAS) between these two groups by employing Student’s t test and subsequently performed FDR correction of the P value. The significance threshold was FDR ≤ 0.05. PAS (high gene A expression) > PAS (low gene A expression) indicated that gene A could activate a pathway. Conversely, PAS (high-gene A expression) < PAS (low-gene A expression) indicated that gene A could inhibit a pathway, as previously described^30,31^.

### Survival analysis

As in previous studies, bioinformatics analyses can be used to reveal potential biomarkers related to survival^32,33^. To begin the process of gene expression and survival analysis, we gathered clinical data from patients who had 33 distinct types of cancer. Patients were excluded from subsequent analyses, such as analyses on overall survival (OS), disease-free survival (DFS), disease-specific survival (DSS), and progression-free survival (PFS), if their data were incomplete or they had comorbidities.

Subsequently, we used sample barcodes to integrate gene expression data with survival data. According to their gene expression levels, patients were included in the HRG or LRG with the median gene expression value as the cutoff point. We used the “survival” R package to determine the survival duration and status of patients in these two groups. Finally, we performed Kaplan‒Meier analysis and employed Cox proportional hazard analysis and a log-rank test to assess the role of each of the selected genes in the prognosis of all cancer types. Genes with log-rank test p values < 0.05 were retained for further analysis.

### Immune cell infiltration analysis

TIMER, the Tumor IMmune Estimation Resource, is a valuable data source specifically created for analyzing the infiltration of immune cells in various types of cancer. It leverages various algorithms to provide insights into immune cell distribution^34^. By utilizing the TIMER 2.0 database (http://timer.cistrome.org/) within the “Gene” function of the “Immune Association” section, we obtained DCBLD2-associated correlations with immune cell infiltration from the TCGA pan-cancer project. Next, we visualized the statistical Spearman’s correlation between DCBLD2 mRNA expression and the infiltration levels of 21 immune cell subsets.

For single-cell resolution analysis, we compared the expression of DCBLD2 and its association with survival and the infiltration of difference cell subtypes across cancers. To achieve this goal, we utilized single-cell datasets collected from the Tumor Immune Single-cell Hub (TISCH) (http://tisch.comp-genomics.org/)^35^.

### Drug sensitivity analysis using the GDSC and CTRP

We acquired IC50 data for 265 small compounds in 860 cellular lines and the corresponding gene expression profiles for these cell lines from the GDSC. Additionally, we collected IC_50_ data for 481 small compounds in 1001 cell lines and the corresponding gene expression profiles of these cell lines from the CTRP. Then, the information regarding gene expression and the drug sensitivity was merged. Finally, Pearson correlation analysis was conducted to determine the correlation between IC_50_ values and gene expression. FDR was used to adjust the p values.

### Pathway exploration at the single-cell level for gene set variation analysis (GSVA)

To obtain a more comprehensive understanding of the functions and pathways associated with iAge-CRGs across cancers, we conducted an extensive search across various databases. One of the databases we utilized was the Cancer Single-Cell State Atlas (CancerSEA) database (http://biocc.hrbmu.edu.cn/CancerSEA/). It is a valuable resource that enable researchers to overcome the challenges posed by tumor heterogeneity.

Our analysis focused on examining the correlations between iAge-CRGs and functional status in various cancer types utilizing data sourced from the CancerSEA database. This approach helped us uncover associations between iAge-CRGs and specific functional states in the context of different cancers, enhancing our understanding of their roles and pathways.

### ICIs and GSVA

To explore the relationship between the expression of iAge-CRGs and the response to immune checkpoint inhibitors (ICIs), we employed the ICI and GSVA score modules. We used ImmuCellAI (http://bioinfo.life.hust.edu.cn/ImmuCellAI/#!/) to assess the infiltration of 24 different immune cell types. The GSVA score, which reflects the integrated gene set expression levels, was utilized.

Patients in the tumor group (TG) had higher GSVA scores than did patients in the normal group, indicating higher overall gene expression. The GSVA score was calculated using the “GSVA” R package. Afterward, we conducted a Spearman’s correlation analysis to assess the correlation between the expression of iAge-CRGs and the response to ICIs. The results included correlation coefficients and p values adjusted based on the FDR. Importantly, the infiltration levels of 24 immune cell types were assessed using marker gene sets, ensuring no overlap between the input genes and the markers during the estimation of the ICI response.

### Construction and validation of the iAge-CRG-related prognostic model

In this study, a risk model was constructed through LASSO Cox regression analysis using prognosis-related iAge-CRGs from the entire dataset (n = 133). Individuals were divided into high-risk and low-risk subgroups based on the median risk score.

To compare survival rates between the high-risk and low-risk groups, survival analyses were conducted based on Kaplan‒Meier survival curves and log-rank tests.

Time-dependent receiver operating characteristic (ROC) curves were used to evaluate the predictive value of the iAge-CRG-associated risk model. To assess the accuracy of the model in predicting patient outcomes, the AUC values for the respective areas under the curve were computed.

To confirm the predictive ability of the iAge-CRG association signature, we conducted both univariate and multivariate Cox regression analyses. Moreover, we developed a nomogram and calibration plots to evaluate the ability of the model to predict the prognosis of UVM comprehensively. This nomogram incorporates clinicopathological factors to provide a more comprehensive prognostic tool. Finally, we explored the relationships between the risk score and OS as well as various clinical characteristics of UVM patients. This was achieved through survival analyses of patient subgroups, which further clarified the relationship between the risk score and patient outcomes and characteristics.

### Associations of the prognostic model score with immune cell infiltration

TIMER was used to assess the impact of the chosen iAge-CRGs in the risk model on the levels of infiltration of six different immune cell types.

### Statistical analysis

The data were analyzed using R software (version 4.2.2). To assess correlations, we utilized Spearman’s correlation analysis. To assess patient survival risk with HR values, we employed a Cox proportional hazard model. We utilized the Wilcoxon test (for 2-stage groups) and ANOVA (for > 2-stage groups) to analyze and compare the GSVA scores of patients across various groups. The trend analysis was conducted using the Mann–Kendall trend test. Student’s t test was used to determine the disparity in the PAS score between the two groups of patients. Unless otherwise specified, the rank-sum test was employed to compare values between two groups. *p* < 0.05 or *FDR* ≤ 0.05 was considered to indicate statistical significance.

## Results

### Analysis of iAge-CRGs and gene mutations

**Figure 1:**
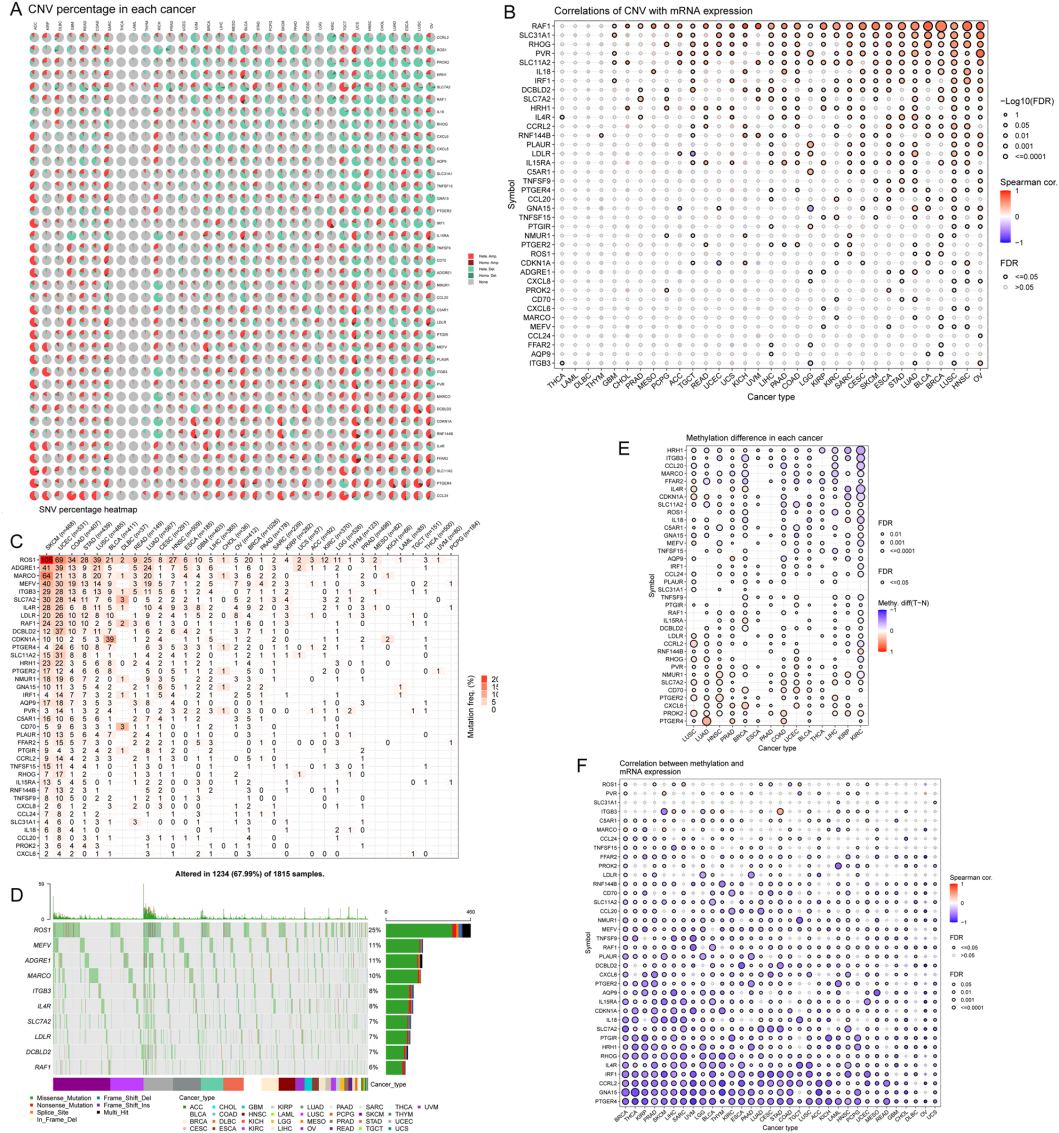
Copy number variation (CNV) distribution, single nucleotide variation (SNV) frequency and methylation distribution of inflammatory aging clock-related genes (iAge-CRGs) in 33 tumors. (**A**) CNV pie chart showing the combined heterozygous/homozygous CNVs of each gene in each cancer. A pie chart representing the proportions of different types of CNVs of one gene in one cancer, where different colors represent different types of CNVs. *Hete Amp* heterozygous amplification, *Hete Del* heterozygous deletion, *Homo Amp* homozygous amplification, *Homo Del* homozygous deletion, *None* no CNV. (**B**) The association between paired mRNA expression and CNV rate in samples was based on Spearman’s product moment correlation coefficient. The size of the point represents the statistical significance, where the larger the dot size is, the greater the statistical significance. (**C**) SNV oncoplot. An oncoplot showing the mutation distribution of iAge-CRGs and a classification of SNV types. (**D**) Mutation frequency of iAge-CRGs. The numbers represent the number of samples that have the corresponding mutated gene for a given cancer. A ‘0’ indicates that there was no mutation in the gene coding region, and no number indicates that there was no mutation in any region of the gene. (**E**) Differential methylation in iAge-CRGs between tumor and normal samples in each cancer type. Blue indicates decreased methylation in tumors, and red indicates increased methylation in tumors; the darker the color is, the larger the difference in methylation level. (**F**) Correlation between methylation and mRNA expression. Blue indicates a negative correlation, and red indicates a positive correlation; the darker the color is, the greater the correlation. All the FDRs of genes and cancer types were less than 0.05 in the figure. FDR, false discovery rate.

In our analysis, we examined copy number variations (CNVs) in iAge-CRGs using data from patients in The Cancer Genome Atlas (TCGA). A pie chart of the CNV distribution revealed that the most common types of CNVs detected in patients were heterozygous amplifications and deletions (Fig. 1A).

Furthermore, we detected a positive correlation between aberrant expression of iAge-CRG and copy number variations (CNVs) in the RAF1 gene among individuals diagnosed with BRCA, BLCA, HNSC, and various other forms of cancer. Our results also indicated a negative correlation between LDLR expression and CNVs in TGCT patients and between GNA15 expression and CNVs in ACC and LGG patients. These correlations were statistically significant (*p* < 0.0001, Fig. 1B). These results indicate that the aberrant expression of iAge-CRGs induced by CNVs might contribute to the progression of cancer.

We also conducted an analysis of single nucleotide polymorphisms (SNPs) in iAge-CRGs to assess the frequency and variants of genes in each cancer subtype. As demonstrated in Fig. 1C, the frequency of SNVs in iAge-CRGs in patients with SKCM, UCEC, COAD, STAD, LUSC, BLCA, LUAD and HNSC ranged from 0 to 105%. We further revealed that 67.99% of patients (1234 out of 1815) exhibited SNVs in regulators (Fig. 1D). Among these SNVs, missense mutations were the most prevalent SNP type in patients. Notably, the top 10 mutated genes, including ROS1, MEFV, ADGRE1, MARCO, ITGB3, IL4R, SLC7A2, LDLR, DCBLD2, and RAF1, accounted for a significant proportion of these mutations, with frequencies ranging from 6 to 25%. Patients with SKCM, LUSC, UCEC, and LUAD had greater frequencies of SNVs in the regulator genes (Fig. 1D).

### Differential methylation analysis of iAge-CRGs across cancers

By investigating the methylation status of these genes, we explored the epigenetic control of iAge-CRGs. The methylation status of the iAge-CRGs exhibited a high degree of patient heterogeneity, as illustrated in Fig. 1E.

Our findings suggest an increase in hypermethylation of iAge-CRGs in patients with LUSC, LUAD, HNSC, PRAD, BRCA, ESCA, COAD and UCEC. In turn, we observed an increase in hypomethylation in iAge-CRGs in patients with KIRC, KIRP, LIHC, THCA, BLCA and PAAD. In most cancers, genes such as PTGER4, PROK2, CXCL6, PTGER2, CD70, SLC7A2, NMUR1, PVR, and RHOG were found to be excessively methylated (*FDR* ≤ 0.05, Fig. 1E). Conversely, HRH1, ITGB3, CCL20, MARCO, FFAR2, IL4R, CDKN1A, SLC11A2, ROS1, IL18, C5AR1, and GNA15 exhibited reduced methylation in most cancers (*FDR* ≤ 0.05, Fig. 1E). Additionally, we analyzed the correlation between methylation status and gene expression. In patients with STAD, LIHC, BLCA, and THYM, there was inverse relationship between the expression of most iAge-CRGs, specifically ITGB, and methylation. However, a positive correlation was observed between ROS1 methylation and its expression in patients with SARC and TGCT. These correlations were statistically significant (*FDR* ≤ 0.05, Fig. 1F).

### Differential expression of iAge-CRGs across cancers and their impact on pathway activity and prognosis

**Figure 2:**
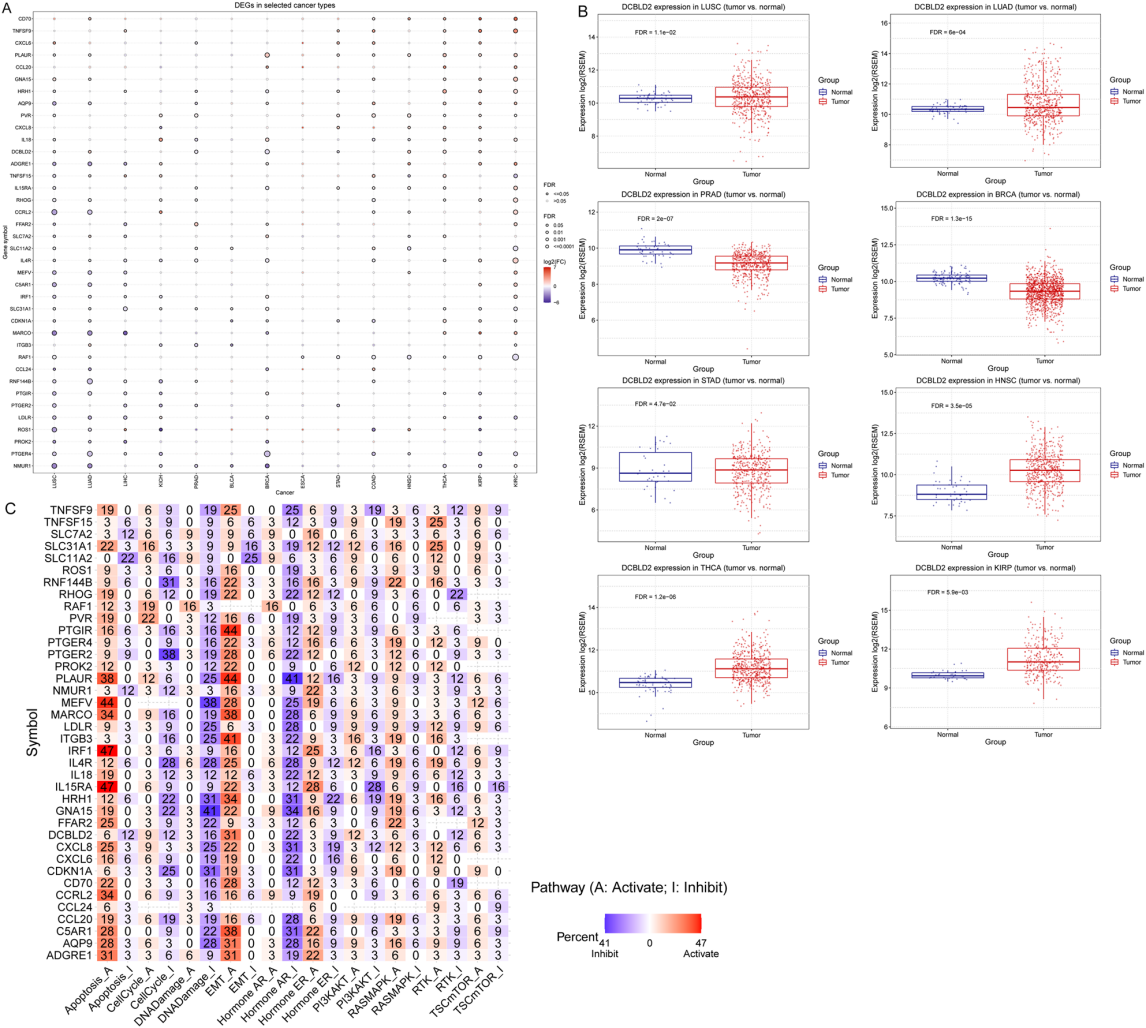
Differential expression of inflammatory aging clock-related genes (iAge-CRGs) across cancers and their impact on pathway activity and prognosis. (**A**) The mRNA differences between normal samples and tumor samples. (**B**) Box plots showing the distribution of DCBLD2 expression across tumor and normal tissue samples from 8 cancer types. (**C**) The combined percentage of the effect of iAge-CRGs on pathway activity.

We explored the differences in iAge-CRG expression among cancer patients. Significant variations in iAge-CRG expression were observed in patients with 13 types of solid tumors, including BLCA, LUAD, BRCA, LUSC, LIHC, PRAD, COAD, KICH, HNSC, KIRC, STAD, KIRP, and THCA (*FDR* ≤ 0.05, Fig. 2A). However, no significant difference in iAge-CRG expression was detected among patients with ESCA. In particular, we noted that DCBLD2 exhibited significantly greater expression in cancer vs. normal samples for 8 cancer types (Fig. 2B).

Pathway activity analysis demonstrated the significant involvement of 38 iAge-CRGs in pathways related to cancer. The pathways included the cell cycle, programmed cell death, TSC/mTOR, RTK, RAS/MAPK, and PI3K/AKT signaling pathways, hormone-related pathways such as ER and AR, EMT, and the reaction to DNA damage (Fig. 2C). The main functions of these iAge-CRGs were related to induction of apoptosis, suppression of cell cycle progression, EMT, the hormones AR and ER, the RAS/MAPK signaling pathway, and the reaction to DNA damage (*p* < 0.05, Fig. 2C).

**Figure 3:**
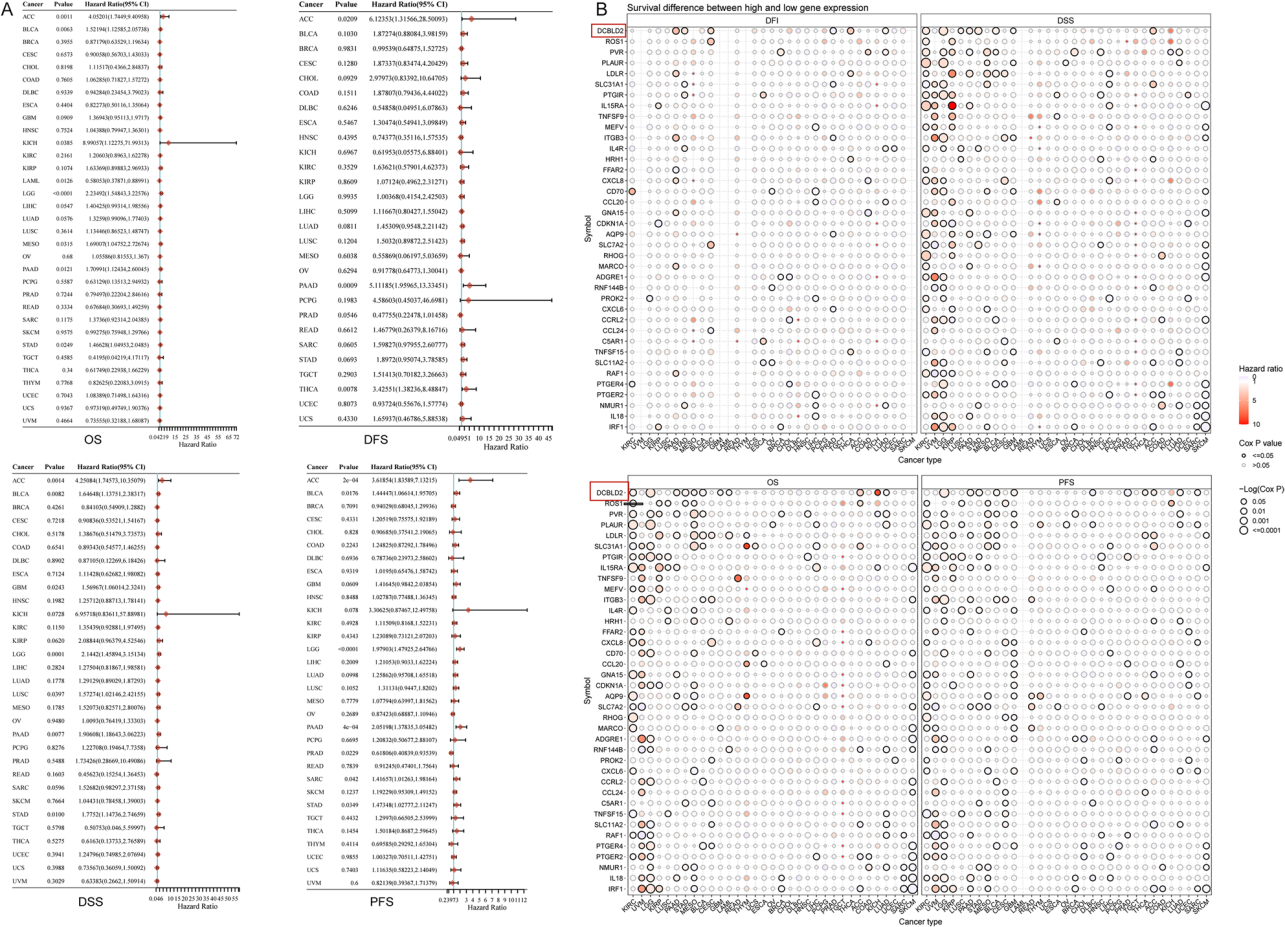
Survival analysis. (**A**) A forest plot of the univariate Cox regression analysis (OS, DFS, DSS, PFS) of DCBLD2 across different cancer types. (**B**) Differences in survival between patients with high and low gene expression. Red points indicate poor survival in the high-expression group, and blue points indicate poor survival in the low-expression group. The size of the point represents the statistical significance, where the larger the dot size is, the greater the statistical significance.

Moreover, a notable association was found between the expression of the iAge-CRG and patient survival status (DFI, DSS, OS, and PFS) (Cox *p* < 0.05, Fig. 3A). These findings suggested that abnormal expression of iAge-CRGs could be implicated in tumorigenesis. Significantly, increased DCBLD2 expression was linked to poorer overall survival in 8 types of cancer, inferior disease-free survival in 3 types of cancer, inferior disease-specific survival in 7 types of cancer, and inferior progression-free survival in 7 types of cancer (Cox *p* < 0.05, Fig. 3B). This finding suggested that DCBLD2 might act as a cancer-causing gene in different types of cancer.

### DCBLD2 is mainly expressed in TAMs in colon cancer

**Figure 4:**
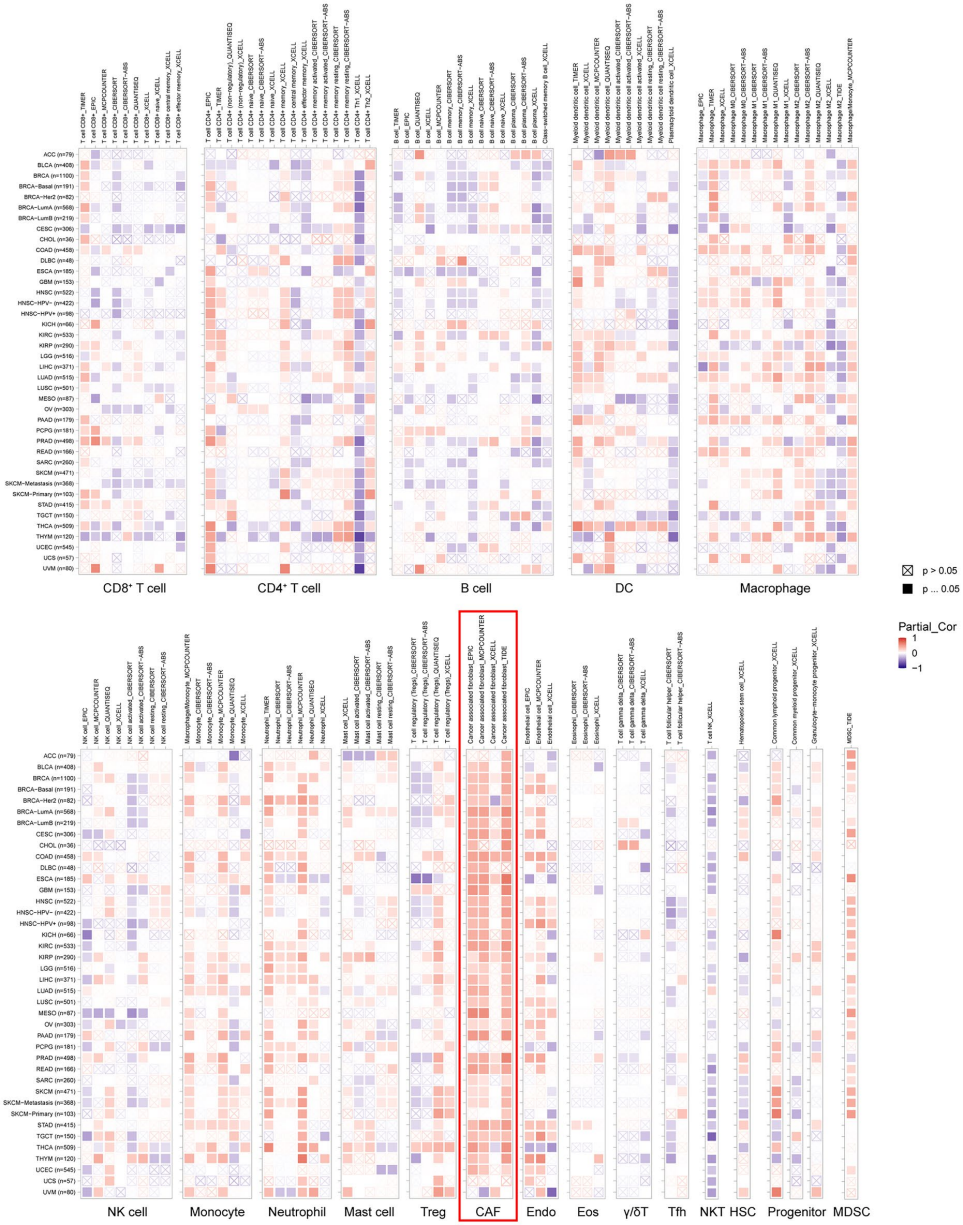
The correlation between DCBLD2 expression and the infiltration of various immune cells in cancers. *DC* dendritic cell, *NK cell* natural killer cell, *Treg* T-cell regulatory, *CAF* cancer-associated fibroblast, *Endo* endothelial cell, *Eos* eosinophil, *g/d T cell* gamma delta T cell, *Tfh* T follicular helper cell, *NKT cell* nature killer T cell, *HSC* hematopoietic stem cell, *MDSC* myeloid-derived suppressor cell.

To determine the immunological components of DCBLD2 in the tumor microenvironment across various cancers, we conducted a comprehensive analysis using TIMER 2.0. This analysis focused on examining the correlation between DCBLD2 expression and immune cell infiltration. As shown in Fig. 4. In most cases, DCBLD2 expression is moderately positive and strongly associated with the quantity of multiple infiltrating immune cells, such as neutrophils, cancer-associated fibroblasts (CAFs), endothelial cells, and myeloid-derived suppressor cells (MDSCs), in different types of cancer. Additionally, it was weakly positively and significantly correlated with the abundance of monocytes and common lymphoid progenitors. Nevertheless, the relationships between DCBLD2 expression and the levels of CD8+ T cells, CD4+ T cells, B cells, DCs, macrophages, NK cells, and Tregs varied depending on the algorithm used. Furthermore, there was a significant inverse relationship between DCBLD2 expression and Th1 cell infiltration in nearly all types of malignancies.

**Figure 5:**
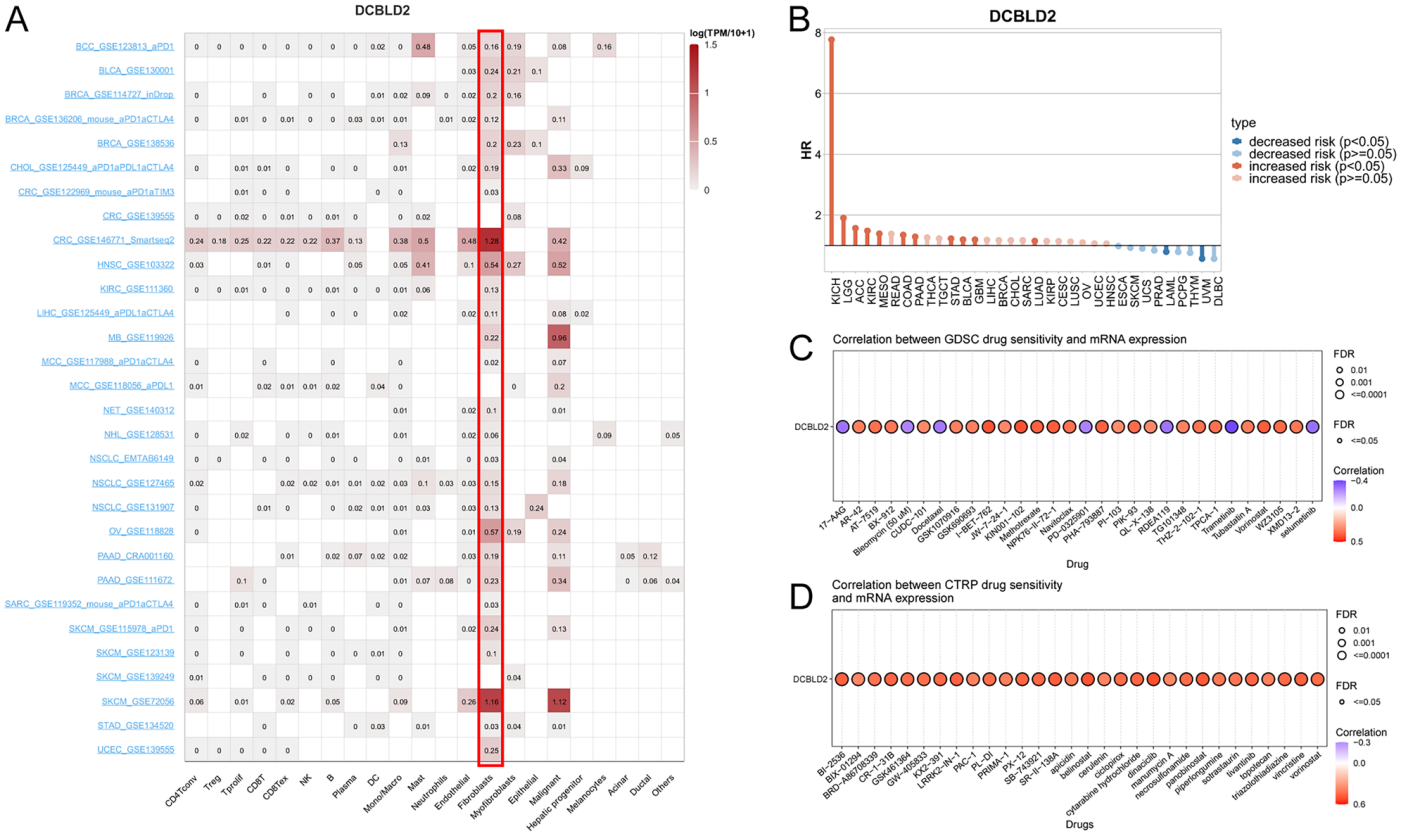
Expression of DCBLD2 in individual cell types and its value in predicting the response to immunotherapy and other drugs. (**A**) DCBLD2 expression in cancer single-cell clusters obtained from the TISCH online tool. (**B**) Survival according to DCBLD2 expression in single-cell clusters obtained from the TISCH online tool. Small compounds predicted to be effective in patients with different DCBLD2 levels according to the CTRP (**C**) and GDSC (**D**) databases.

Notably, the presence of CAFs is strongly correlated with the expression of DCBLD2 in various cancer types. More importantly, we retrieved single-cell DCBLD2 expression data from TISCH. As shown in Fig. 5A, DCBLD2 is expressed by fibroblasts in BCC, BLCA, BRCA, CHOL, CRC, HNSC, KIRC, LIHC, MCC, MB, NET, NHL, NSCLC, OV, PAAD, SARC, SKCM, STAD, UCEC, and SARC. Moreover, high DCBLD2 expression was correlated with worse survival in KICH, LGG, ACC, KIRC, MESO, COAD, PAAD, STAD, BLCA, GBM, and LUAD patients (*p* < 0.05, Fig. 5B).

Genetic abnormalities affect a patient’s sensitivity to chemotherapy and targeted therapies. We therefore investigated the involvement of DCBLD2 in mediating patient response to chemotherapy or targeted therapy. We first integrated cancer cell drug sensitivity and gene expression data from the GDSC. Spearman’s correlation analysis revealed a negative correlation between the expression of DCBLD2 and sensitivity to various drugs, including 17-AAG, bleomycin (50 µM), docetaxel, PD-0325901, RDEA119, trametinib, and selumetinib, as indicated by the IC_50_ values. However, the expression of DCBLD2 was positively correlated with the IC_50_ values of AR-42, AT-7519, BX-912, CUDC-101, GSK1070916, GSK690693, I-BET-762, JW-7-24-1, KIN001-102, methotrexate, NPK76-II-72-1, Navitoclax, PHA-793887, PI-103, PIK-93, QL-X-138, TG101348, THZ-2-102-1, TPCA-1, tubastatin A, vorinostat, WZ3105, and XMD13-2 (*FDR* ≤ 0.05, Fig. 5C). Spearman’s correlation analysis revealed that high expression of DCBLD2 was correlated with resistance to various drugs, such as BI-2536, BIX-01294, BRD-A86708339, CR-1-31B, GSK461364, GW-405833, KX2-391, LRRK2-IN-1, PAC-1, PL-DI, PRIMA-1, PX-12, SB-743921, SR-II-138A, apicidin, belinostat, cerulenin, ciclopirox, cytarabine hydrochloride, dinaciclib, manumycin A, necrosulfonamide, panobinostat, piperlongumine, sotrastaurin, tivantinib, topotecan, triazolothiadiazine, vincristine, and vorinostat (positive correlation with IC_50_) (*FDR* ≤ 0.05, Fig. 5D). These findings indicate that the abnormal expression of DCBLD2 might be responsible for resistance to chemotherapy and targeted treatment.

### Analysis of the function of the iAge GSVA score at the single-cell level

**Figure 6:**
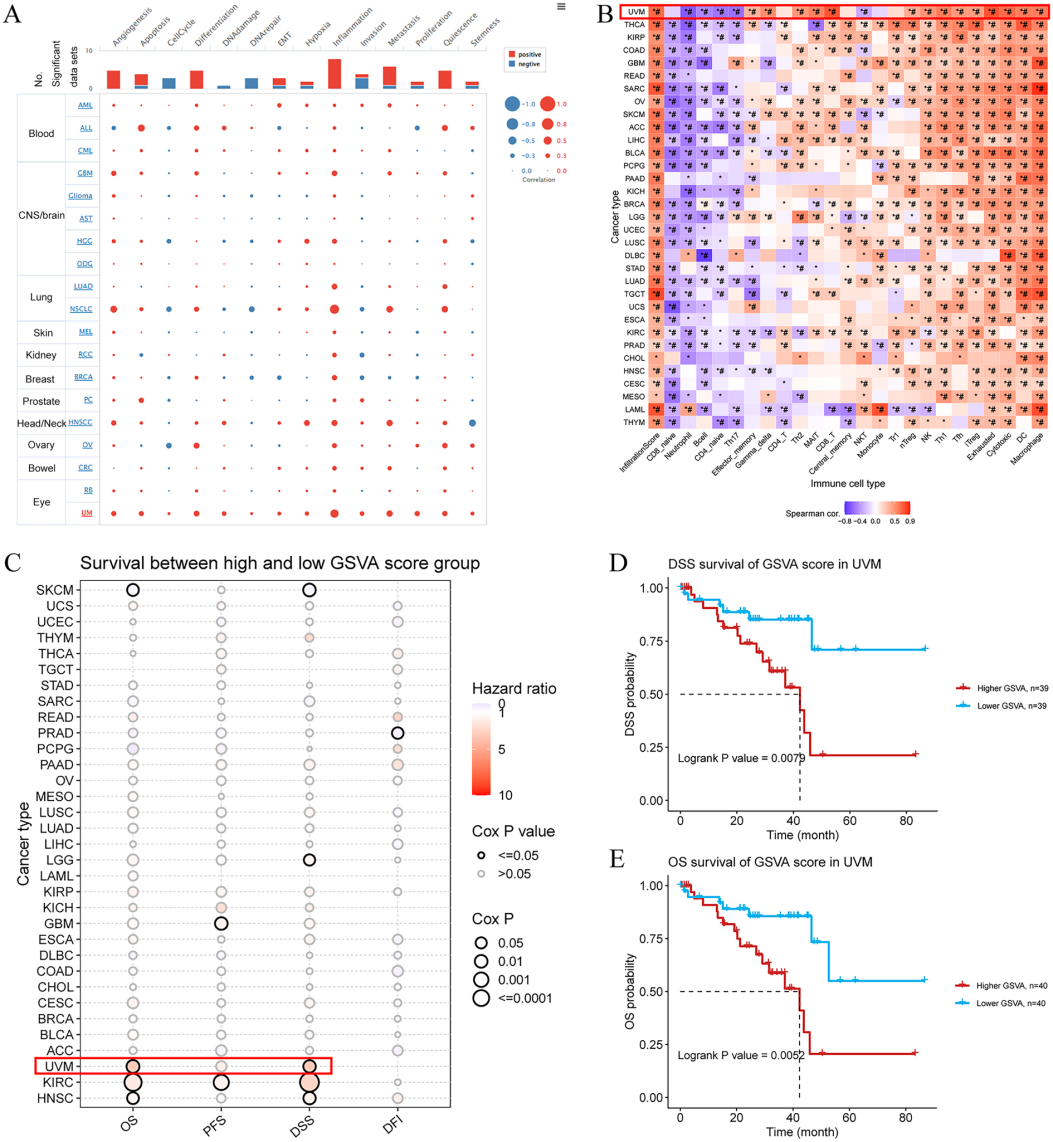
The inflammatory aging (iAge) gene set variation analysis (GSVA) enrichment scores. (**A**) Correlation of iAge GSVA scores with 14 functional states in patients with different tumors. Red represents a positive correlation, and blue represents a negative correlation. (**B**) Correlations between GSVA enrichment scores of iAge-related genes and immune cell infiltration in 33 cancers. *p value ≤ 0.05; ^#^FDR ≤ 0.05. (**C**) Survival between the high and low GSVA score groups. (**D**) Disease-specific survival (DSS) and (**E**) overall survival (OS) of patients with UVM according to the GSVA score.

We analyzed the correlation between the iAge GSVA and 14 functional states in different tumors using CancerSEA data. The iAge GSVA scores showed a positive correlation with inflammation in AML, ALL, CML, GBM, glioma, AST, HGG, ODG, LUAD, NSCLC, MEL, RCC, BRCA, PC, HNSC, OV, CRC RB, and UVM (p < 0.05). In UVM, the iAge GSVA scores were positively correlated with angiogenesis, apoptosis, the cell cycle, differentiation, DNA repair, EMT, hypoxia, inflammation, invasion, metastasis, proliferation, quiescence, and stemness (*p* < 0.05, Fig. 6A).

Moreover, we established a notable association between GSVA enrichment scores (ESs) of iAge-CRGs and the IC50s of ICIs in 33 different types of cancer (with p values and FDRs ≤ 0.05, as shown in Fig. 6B). Furthermore, the findings indicated a positive correlation between the GSVA ESs of iAge-CRGs and the levels of macrophages, DCs, cytotoxic and depleted T cells, and iTreg, Tfh, Th1, and NK cells. Furthermore, we detected an inverse relationship between the GSVA ESs of iAge-CRGs and the levels of naïve CD8+ and CD4+ T cells, neutrophils, and B cells across various types of cancer. On the other hand, in the pan-cancer dataset, the GSVA ESs of the iAge-CRGs were positively correlated with the infiltration score of the patients. As a result, aberrant iAge-CRG expression modulates the response to ICIs in patients, suggesting a significant role for iAge-CRG in cancer progression, particularly UVM progression.

To investigate the association between the expression of iAge-CRGs and the survival of patients with tumors, we analyzed OS, PFS, DSS, and DFI in patients grouped according to iAge-CRG scores. The prognostic value of the iAge-CRGs was assessed through univariate Cox regression analysis (Fig. 6C). Overall, a high iAge-CRG score in the SKCM and PCPG cohorts indicated a good prognosis and was a protective factor for patients. KIRC, HNSC, and UVM patients with a high iAge-CRG score exhibited a poor prognosis (Fig. 6E), and this variable was identified as a risk factor (all *p* < 0.05). The PFS results indicated that an elevated iAge-CRG score was associated with a poor prognosis in KIRC and GBM patients and served as a risk factor for patients (all p < 0.05). The DSS results indicate that a high iAge-CRG score in SKCM and PCPG patients indicated a good prognosis and was a protective factor for patients. A high iAge-CRG score was associated with a poor prognosis in KIRC, UVM (Fig. 6D), HNSC, and LGG patients and was a risk factors for patients (all *p* < 0.05). The DFI results showed that a high iAge-CRG score indicated a good prognosis in patients with PRAD and was a protective factor for patients. A high iAge-CRG score was associated with a poor prognosis in the MESO and KICH cohorts and was identified as a risk factor for patients (all *p* < 0.05). The findings indicate that the iAge-CRG score can be used as a conventional prognostic predictor and can assist in predicting outcomes in patients with various types of tumors, especially UVM.

### Construction of a prognostic risk model based on of iAge-related genes in UVM

**Figure 7:**
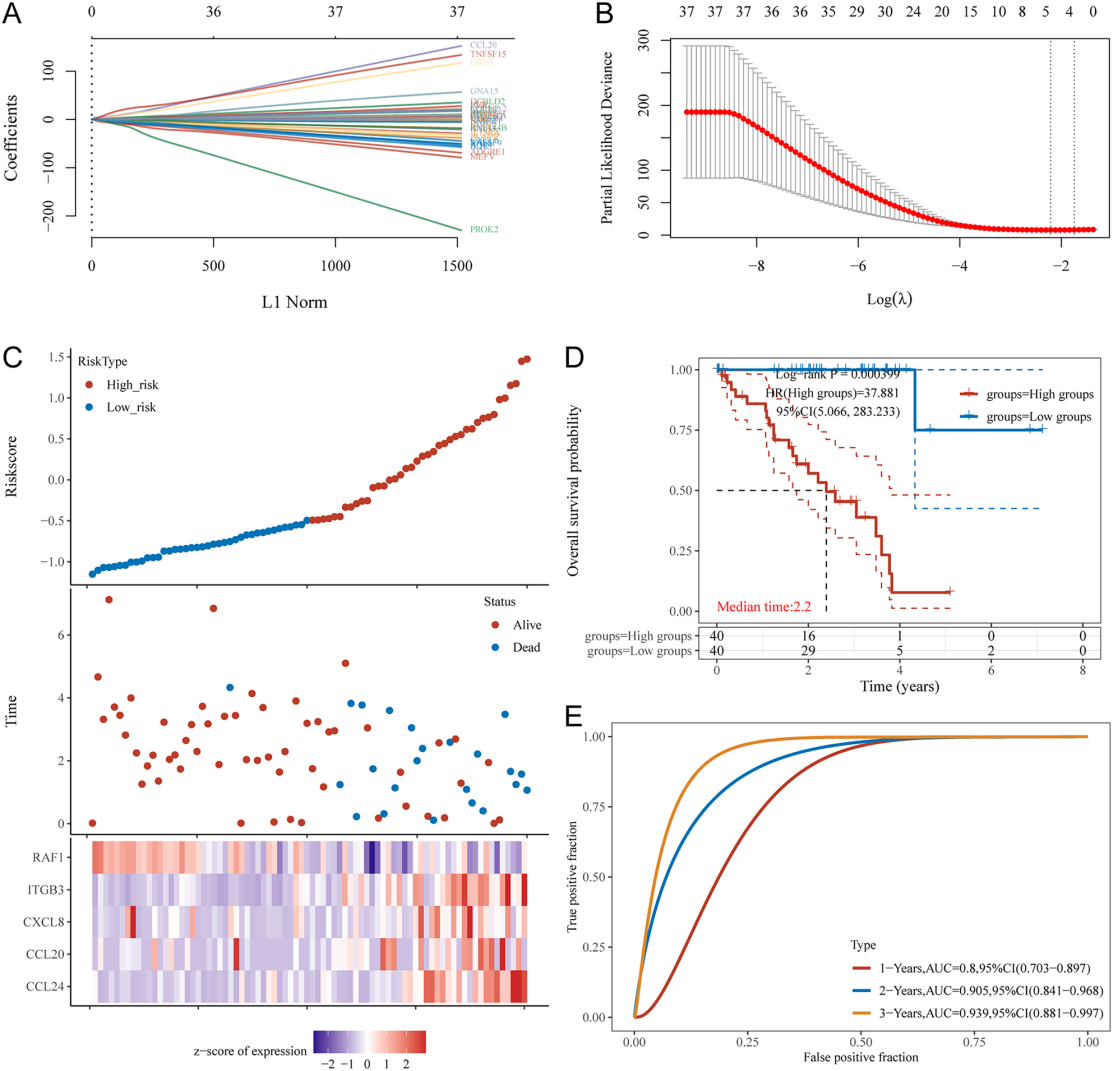
Construction of a prognostic risk model of inflammatory aging clock-related genes (iAge-CRGs) for UVM patients. (**A**) The trajectory of each independent variable. The horizontal axis represents the log value of the independent variable lambda, and the vertical axis represents the independent variable’s coefficient. (**B**) Confidence intervals with different values of lambda. (**C**) Risk plot distribution, survival status of patients, and heatmap of the expression of the 5-gene signature in the whole TCGA-UVM dataset. (**D**) Kaplan–Meier survival curves for the risk model. (**E**) ROC curves for the risk model.

LASSO Cox regression analyses was used to remove iAge-CRGs that were falsely predicted as having a positive effect on prognosis. The obtained iAge-CRGs, shown in Fig. 7A,B, included ITGB3, CXCL8, CCL20, RAF1, and CCL24. Using LASSO Cox regression analysis, prognostic risk models were built based on the five chosen iAge-CRGs. This analysis revealed that ITGB3, CXCL8, CCL20, and CCL24 are risk factors, whereas RAF1 is a protective factor. The risk score formula was as follows: risk score = (0.374) × CCL24 + (0.9113) × CCL20 + (0.094) × CXCL8 + (0.6444) × ITGB3 + (− 0.3083) × RAF1.

Subsequently, individuals were categorized into high-risk and low-risk subgroups according to the median risk score. Figure 7C shows the distribution of risk plot and survival status among UVM patients; the results revealed significantly lower OS rates for high-risk patients than for low-risk patients. Furthermore, the gene heatmap revealed that the levels of ITGB3, CXCL8, CCL20, and CCL24 were markedly elevated in the high-risk subgroup, whereas the expression of RAF1 was notably increased in the low-risk subgroup (Fig. 7C). Ultimately, five iAge-CRGs, namely, ITGB3, CXCL8, CCL20, RAF1, and CCL24, were shown to have prognostic significance for OS. These findings were subsequently utilized to construct prognostic risk models. The prognostic value of the iAge-CRG signature was confirmed using the entire dataset consisting of 133 samples. Kaplan‒Meier survival analyses indicated that patients in the high-risk group experienced shorter OS than did those in the low-risk group (*p* = 0.000399; Fig. 7D). In the training cohort, the ROC curves for the iAge signature had AUC values of 0.8, 0.905, and 0.939 for 1 year, 2 years, and 3 years, respectively (Fig. 7E). Collectively, these results indicate the precision of the UVM prognostic risk model based on the four chosen iAge-CRGs.

**Figure 8:**
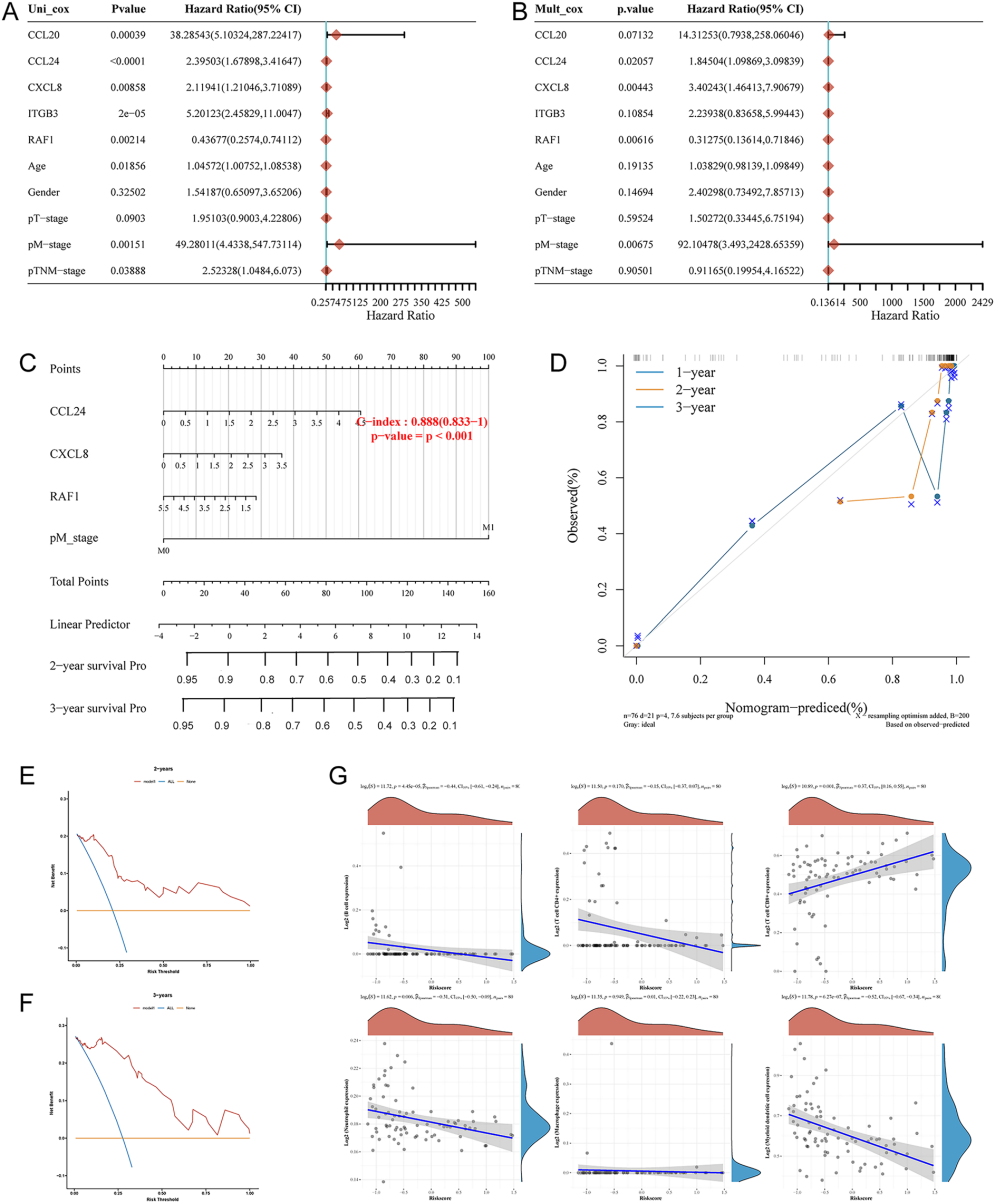
Relationships between the risk model and overall survival rate, as well as clinicopathological features, among patients diagnosed with UVM. (**A**) Univariate and (**B**) multivariate Cox analyses of clinical characteristics. (**C**) The nomogram based on pathological M stage for predicting the prognosis of UVM patients. (**D**) Calibration plots for the overall survival nomogram model. The dashed diagonal indicates the ideal nomogram, and the purple line, the orange line, and the blue line represent the predicted 1-year, 2-year, and 3-year overall survival, respectively, of UVM patients. Decision curve analysis of candidate mRNAs for predicting (**E**) 2-year and (**F**) 3-year survival status. (**G**) The correlations between gene expression and immune score were analyzed via Spearman’s correlation analysis.

Univariate Cox proportional hazards regression analyses were performed to assess the prognostic significance of differentially expressed iAge-CRGs in UVM patients. The aim of this analysis was to determine the association between the mRNA expression levels of the iAge-CRGs and OS in UVM patients in the entire TCGA dataset (n = 133). We assessed the predictive value of iAge-CRGs that were differentially expressed in UVM patients by conducting univariate Cox proportional hazards regression analyses on the entire TCGA dataset, which consisted of 133 patients. The OS of UVM patients was significantly associated with the levels of the 5 differentially expressed iAge-CRGs (CCL20, CCL24, CXCL8, ITGB3, and RAF1) (*p* < 0.05, Fig. 8A). Subsequently, the influence of three iAge-CRGs (CCL24, CXCL8, and RAF1), on the OS and clinical outcomes of UVM patients was assessed using multivariate Cox regression analyses (*p* < 0.05, Fig. 8B). In addition, univariate Cox regression analysis revealed a significant association between OS and age, pathological M stage, and pathological TNM stage in UVM patients (*p* < 0.05, Fig. 8A). Multivariate Cox regression analyses demonstrated a clear association between pathological M stage and OS in UVM patients (*p* < 0.05, Fig. 8B). A nomogram (Fig. 8C) was constructed to predict the prognosis of patients with UVM by incorporating pathological M stage with the risk score and achieved a C-index of 0.888 (p < 0.001) for predicting survival. The calibration curves of the nomogram model for 1, 2, and 3 years demonstrated strong agreement between the values predicted by the nomogram and the observed outcomes (Fig. 8D). Decision analysis based on curves (DCA) revealed that UVM can be effectively diagnosed according to the expression of candidate mRNAs, and this model had a net benefit over the “all-patients-died” and the “no-patients-died” models (Fig. 8E,F). The combined prognostic nomogram, which relies on the iAge-CRG signature, demonstrated outstanding accuracy in predicting the overall survival of UVM patients.

### Associations of the risk model with the immune cell infiltration level in UVM

Correlation analysis was performed to assess the impact of the four-iAge-CRG risk model on the tumor immune microenvironment (TIME) for UVM patients by examining the relationship between the risk score and infiltration levels of six different types of immune cells (Fig. 8G). The findings indicated that there was an inverse association between the risk score and the levels of B cells (p < 0.001), neutrophils (p < 0.05), and myeloid dendritic cells, while a positive correlation was observed between the risk score and the level of CD8+ T cells (*p* < 0.05). However, no significant negative correlation was detected between the risk score and the infiltration level of CD4+ T cells (*p* > 0.05), macrophages (*p* > 0.05), or myeloid dendritic cells (*p* > 0.05).

## Discussion

During the process of tumorigenesis, cells undergo many changes and acquire mutations. Recent research indicates a significant increase in the levels of specific inflammatory components as individuals age, particularly in connection with the development of cancers^19,20,36^. It is therefore likely that age may be significantly involved in inflammation and cancer initiation. In this study, the iAge model was created using an algorithm comparable to that in a previous publication^37^. It is necessary to identify the major iAge contributors. Unlike studies on other previously defined cancer-related clocks^38,39^, studies on the iAge clock could aid in identifying the features of inflammation and explaining the complex correlation between chronic inflammation and age. Hence, the examination of iAge-CRGs has the potential to improve our understanding of cancer and pinpoint possible strategies for the treatment of cancer patients. Given the intricate connections among age, inflammation, and cancer, we conducted a thorough examination of the genetic, immune, and clinical features associated with iAge-clock-related genes (CRGs) across 33 different types of cancer.

In total, 38 iAge-CRGs were identified. One study showed a correlation between iAge-CRGs and cardiovascular disease risk in adults^28^. However, no studies have been published on the use of the iAge clock in guiding cancer treatment. Furthermore, the correlations of iAge-CRG expression and the immunological and clinical characteristics in 33 cancer types patients have not been assessed.

According to our analysis, the CNV rate of the iAge-CRGs was significantly higher in tumor samples vs. normal samples. We observed a direct relationship between CNV and the expression of iAge-CRGs, suggesting that CNV could impact the expression of iAge-CRGs, potentially influencing tumorigenesis and patient prognosis. Next, we analyzed epigenetic changes and showed that aberrant hypermethylation can reduce the expression of the iAge-CRGs. A study revealed CDKN1A hypomethylation in patients with 11 types of cancer^40^. Furthermore, CXCL8 expression was altered in patients with non-small cell lung cancer (NSCLC)^41^ and colorectal adenocarcinoma^42^ due to aberrant methylation. Studies have shown a correlation between high PTGER2 methylation and neuroblastoma^43^, NSCLC^44^, and cervical cancer progression^45^. Based on our investigation, SNV occurrence in iAge-CRGs appears to be significantly frequent. Additionally, a notable association between SNVs and the expression of iAge-CRGs was identified, suggesting that SNVs may impact the expression of iAge-CRGs. This may contribute to tumorigenesis and survival. One study showed that C5AR1 was frequently mutated in patients with SKCM and UCEC^46^. Therefore, we hypothesize that genetic and epigenetic alterations in iAge-CRGs may lead to dysregulation of iAge-CRGs and thus promote tumorigenesis.

Next, we assessed the expression, related pathways, and survival implications of iAge-CRGs in various tumor types from the TCGA dataset. Our research revealed that iAge-CRGs are expressed in a wide range of tumor types, and these genes, especially DCBLD2, are notably upregulated in most tumors. Pathway analysis revealed that these iAge-CRGs can control pathways associated with cancer. They are involved in pathways related to induction of apoptosis, suppression of cell cycle progression, EMT, the hormones ER and AR, the RAS/MAPK signaling pathways, and the response to DNA damage^47–55^. The findings indicate that iAge-CRGs create a web of pathways associated with cancer, which helps to slow cancer progression and enhance patient survival. To further clarify the role of iAge-CRGs in clinical risk stratification, an evaluation was conducted to examine the correlation between iAge-CRGs and survival. Survival analysis indicated that the overexpression of iAge-CRGs was linked to OS, DFS, DSS, and PFS. Notably, a high level of iAge-CRG expression was associated with an unfavorable prognosis in the majority of tumors. According to these results, we further analyzed immune infiltration in different TCGA tumor types grouped according to DCBLD2 expression.

The concept of cancer immunoediting was introduced by Dunn et al. in 2002^56^. It involves three processes, namely, elimination, equilibrium, and escape, which are dependent on various immune cells present in the tumor microenvironment. Using TIMER 2.0, we conducted a comprehensive analysis across different types of cancer and discovered a strong positive correlation between DCBLD2 expression and the levels of infiltrating immune cells. Notably, this association was particularly prominent for cancer-associated fibroblast (CAF) populations. In this study, the expression of DCBLD2 was found to be positively correlated with the expression of CAFs, which facilitate tumor growth, angiogenesis, invasion, metastasis, and extracellular matrix remodeling^57^. To be more precise, CAFs engage in environmental restructuring, a process that is confirmed through the analysis of either bulk or single-cell transcriptional sequencing information. Nonetheless, we found that DCBLD2 is a reliable biomarker of CAF infiltration. To the best of our knowledge, this study is the first to demonstrate a distinct connection between cancer immunity and iAge-CRGs including DCBLD2. Hence, although further proof of the role of DCBLD2 in CAFs is needed, our research indicates that targeting DCBLD2 signaling in CAFs holds promise as an innovative treatment strategy. Nevertheless, our findings also demonstrated that the expression of DCBLD2 was moderately positively correlated with that of MDSCs, which inhibit the immune response by suppressing the activity of T cells^58^, and that DCBLD2 expression was negatively correlated with the levels of Th1 cells. This discovery could partly explain the protective effect of DCBLD2 in certain forms of cancer.

We identified potential drug modulators of DCBLD2. Thus, our hypothesis is that targeting DCBLD2 could emerge as an optimal approach for the treatment of cancer patients. Nevertheless, further investigations are needed to elucidate the mechanisms underlying the effects of drugs on DCBLD2 expression and cancer progression. Additionally, we further examined the correlation between iAge-CRGs and the immune response against tumors. The iAge-GSVA score is positively correlated with inflammation in most tumors according to single-cell sequencing data. In UVM, the iAge-GSVA score is positively correlated with angiogenesis, apoptosis, the cell cycle, differentiation, DNA repair, EMT, hypoxia, inflammation, invasion, metastasis, proliferation, quiescence, and stemness. Additionally, subsequent investigations revealed a notable and positive correlation between the GSVA ESs of iAge-CRGs and the infiltration score of individuals with UVM. Furthermore, research has indicated a connection between CCRL2, SLC31A1, and ICI in relation to cancer^59,60^. These results suggest that iAge-CRGs can be used to predict the efficacy of immunotherapies in cancers, particularly UVM, which was further demonstrated by survival analyses.

Using LASSO Cox regression analysis, a risk signature consisting of five genes was constructed. The risk model of the four chosen iAge-CRGs was verified as a reliable standalone prognostic factor for predicting clinical outcomes in UVM patients. According to the risk model, ITGB3, CXCL8, CCL20, and CCL24 function as risk factors, whereas RAF1 functions as a protective factor.

Clinical relevance analysis confirmed that the risk score is strongly correlated with the pathological M stage of the tumor. Significantly, survival analysis by subgroup demonstrated that the risk signature based on five genes possesses precise prognostic value for multiple UVM subgroups categorized by age and pathological TNM stage. The calibration curves of the nomogram models for 2-year and 3-year OS demonstrated strong concordance between the values predicted by the nomogram and the observed outcomes. Significantly, the AUC values for the five-gene signature ROC curves at 1, 2, and 3 years surpassed 0.8. The findings indicate that the risk model consisting of five genes has reliable prognostic significance for UVM. To examine the impact of iAge-CRGs on the TIME, we investigated the relationships between the four chosen iAge-CRGs and the extent of immune cell infiltration. To assess the impact of the four iAge-CRG risk models on the TIME in UVM patients, we analyzed the correlation between the risk score and infiltration levels of six immune cell types. The findings indicated that there was an inverse relationship between the risk score and the levels of B cells, neutrophils, and myeloid dendritic cells, while there was a positive correlation between the risk score and the level of CD8+ T cells. However, no significant negative correlation was detected between the risk score and the infiltration levels of CD4+ T cells, macrophages, or myeloid dendritic cells.

Our study has several limitations. First, the main limitation is that dynamic analysis of iAge-CRGs expression in paired tumor samples is difficult at the temporal level, and validation studies are needed. Transcriptomic data from patients were obtained from the TCGA to determine the association between iAge-CRGs and cancer; however, these results should be verified experimentally. However, our findings offer fresh perspectives on the control of iAge-CRGs in cancer. Next, we analyzed the differences in iAge-CRGs at the genetic, epigenetic, expression, and pathway levels. These differences may result in differences in treatment response and prognosis. Therefore, it is necessary to conduct a thorough examination of the diversity of cancers and tailor treatments accordingly.

## Conclusions

In summary, iAge-CRGs, particularly DCBLD2, may be potential targets for improving immunotherapy outcomes. Furthermore, notable associations were detected between aberrant DCBLD2 expression and CAF infiltration and overall survival in patients with various types of cancer. In conclusion, the risk signature consisting of five genes has the potential to serve as an independent prognostic indicator for patients with UVM and plays a significant role in the immunosuppression of UVM.

### Supplementary Information

Supplementary Information.

## Data Availability

Data are provided within the manuscript or Supplementary Information files.
