# Supplementary material for: Comprehensive pan-cancer analysis of inflammatory age-clock-related genes as prognostic and immunity markers based on multi-omics data
Source: Sci Rep. 2024 May 7;14:10468. doi: 10.1038/s41598-024-61381-z (PMC11076581; doi:10.1038/s41598-024-61381-z)
Supplement: Supplementary file 1 — Supplementary Information. [file 41598_2024_61381_MOESM1_ESM.doc]

**Supplementary Materials:**

**Table S1.** The gene signature of 24 immune cells.

| **24 immune cells** | **gene signature** |
| --- | --- |
| Dendritic cells | BLVRB/C1QA/C1QB/CA8/CALY/CRTAM/CSF1R/CXCR3/CYP4F3/FGL2/GATA3/GZMB/IL21R/ITIH4/KCNA5/KCNC3/KCTD5/LILRB4/LMAN2L/NFKB1/PLD2/PRR5L/PTCRA/PTGIR/SIGLEC1/SLAMF8/SLC15A3/ST8SIA1/SYT17/TNFRSF10C/VAV2 |
| B cells | AFTPH/AHSP/ANXA3/BLK/CA1/CD19/CD37/CD79A/CD79B/CNOT1/COL19A1/GNG3/HLA-DPB1/HLA-DQA1/HRH4/LSM6/MEFV/MS4A1/NMUR1/PADI4/PLIN1/PYGM/SGCA/STRN4/TCL6/TRAF3/WNT16 |
| Monocytes | ADCY8/APOC3/CA1/CLCA4/CLEC10A/CPA2/CYBB/CYP4F3/DPP6/FOLR2/GZMH/HSPB6/IGSF6/IL7R/KLRB1/KLRF1/KLRK1/KNG1/LTBR/MAP3K2/NUP214/OSBPL11/RTN3/SERP1/SGCA/SOCS3/TBK1/TLR8 |
| Macrophages | ARPC4/ATP6V0E1/BPI/C1QA/C1QB/CAMP/CHIT1/CLEC5A/CLIP1/CSF1R/CYBB/FGR/GGA1/GRB2/IFNAR1/IGSF6/IL17RA/LILRA2/MARCO/MMP8/MS4A6A/OTUD4/PSME1/RENBP |
| Natural killer (NK) cells | C1QB/CALY/CD37/CLEC10A/CRTAM/CSF2RB/CX3CR1/DNAJB14/GZMB/IL18RAP/IL2RB/IL7R/KIR2DL3/KIR3DL2/KNG1/MAN1C1/NCR1/NCR3/PRR5L/PSMD4/SPON2/SYT17/XCL1 |
| Neutrophils | AATK/ALPL/BMX/BTNL8/CASP5/CEACAM3/CLC/CREB5/CSF2RB/CSF3R/CXCR1/CXCR2/FBXO38/FCGR3B/FPR2/HSPA6/IL18RAP/MAK/MEFV/PADI4/PAK2/S100A12/SIGLEC5/TOP1/TREML2/TRPM6/UBXN2B/VNN3 |
| CD4 T cells | AAK1/CCR4/CD4/CD40LG/DLEC1/GPR171/GPR183/ITIH4/ITK/KLHL3/LAX1/LY9/NCK2/NT5E/PLCG1/PLXDC1/SELL/TNFRSF4/TRMT2B/ZAP70 |
| CD8 T cells | ARHGEF1/CD27/CD7/CD8A/CD8B/CLUAP1/CRTAM/CTSW/CX3CR1/EEF1D/FBXW4/FKTN/GZMH/KLRG1/LY9/PLCG1/PPP1R2/RING1/SF1/SIRPG/TSPAN32/TTN/ZNF611); NKT (CASP5/DOLK/GMIP/PRR5L/SGCA/SLAMF1/TCOF1/TGFBR2 |
| Gamma delta T cells | ACD/C1orf61/CCR5/CHST12/CYP4A11/GNLY/GZMA/GZMB/GZMH/KLRG1/LAG3/LCP2/NKG7/PRF1/PSTPIP1/PTPN4/PVRIG/RALY/TAB2/TDP1/TINF2/ZMAT5 |
| CD4 naive T cells | CD2/CD3G/CD4/CD40LG/GIMAP6/GLG1/HMOX2/IL7R/ITK/LIMD2/LY9/NAA16/OBSCN/PACS1/PLCL1/RPL14/SEPT9/SNPH/TPP2/TRAF1/ZBTB40 |
| CD8 naive T cells | BLNK/CA14/CALY/CCDC87/CCR7/CD8A/CD8B/CRTAM/FXYD7/GJB4/GPR15/HTR1B/KERA/KRT1/LIN28A/MAN1C1/MOGAT2/NKTR/PSG11/RRH/SLC17A4/SMCP/SMR3B/TNKS2/TREM1/ZNF208 |
| Cytotoxic T cells | BLNK/CD8A/CD8B/GNLY/GZMA/GZMH/ITGAM/KLRK1/KNG1/PRF1/PSORS1C2/PTGDR2/SCN3A/TNFRSF10C |
| Exhausted T cells | ADGRG1/AFAP1L2/CCND2/CD38/CD8A/CD8B/CHST12/CTLA4/DFNB31/EOMES/FUT8/ITM2A/LAG3/MYO1E/NDFIP2/PARK7/PDCD1/SIRPG/SNX9 |
| Type 1 regulatory T cell (Treg) | CCR4/CD28/CD4/LAX1/TNFRSF4 |
| Natural Treg | CD4/CD5/CTLA4/DUSP4/FOXP3/IL10RA/IL2RA/SIT1/STAT5A/TNFRSF9 |
| Induced Treg (iTreg) | ATG2B/CCR3/CCR4/CCR8/CD28/CD5/CTLA4/FASLG/FOXP3/GALNT8/HS3ST3B1/ICOS/IL10RA/NFATC3/PPM1B/SIT1/STAT5A/TTN/ZFYVE9 |
| T helper (Th) type 1 | APBB2/CCL4/CTLA4/EIF2B2/GGT1/IFNG/IL2/LTA/MNAT1/SLAMF1/STAT1/SYNGR3/TACO1 |
| Th2 | GATA3/GSTA4/GZMK/IL4/SLC25A44 |
| Th17 | CD4/IL17RA/IL1R1/IL21/RORC |
| T follicular helper cells (Tfh) | CA8/CD2/CD3G/GZMM/ITK/KLRB1/LTA/MAP4K1/ST8SIA1/TRAC/TRAV9-2/TRIB2/UBASH3A |
| Central memory T cells | ADSL/APBB1/ARID5B/ATM/CD247/CD3E/CD40LG/CD5/CDKN2AIP/CORO7/CYLD/DVL1/GIMAP4/GMEB2/GPR171/IL7R/IPCEF1/ITK/LRIG2/LY9/NAA16/NCK1/PURA/RPP38/SNRPN/SPTAN1/TFAP4/TPR/TRADD/UBASH3A |
| Effector memory T cells | APBA3/CD160/CD2/CD8B/CDK10/CDKN2AIP/CHST12/COG4/CX3CR1/DHX16/EWSR1/GIMAP6/GPR171/GZMK/HMOX2/IKZF3/ITK/KLRD1/KLRG1/MAPKAPK5/MORC2/MRFAP1L1/PLCG1/PSMC5/RNF167/SBF1/SF3B2/SLAMF1/TBCD/USP47/ZFYVE9/ZNF549 |
| Mucosal associated invariant T cells | CD8A/CD8B/CERK/DKK3/FLT4/GPR171/NR1D1/SLAMF1/TBC1D31/TC2N |

**Table S2.** Samples of 33 cancer types.

| **33 cancer types** | **Sample size** |
| --- | --- |
| Acute myeloid leukemia (LAML) | n = 191 |
| adrenocortical carcinoma (ACC) | n = 92 |
| bladder urothelial carcinoma (BLCA) | n = 464 |
| breast invasive carcinoma (BRCA) | n = 1218 |
| cervical squamous cell carcinoma and endocervical adenocarcinoma (CESC) | n = 331 |
| cholangiocarcinoma (CHOL) | n = 46 |
| colon adenocarcinoma (COAD) | n = 458 |
| esophageal carcinoma (ESCA) | n = 212 |
| glioblastoma (GBM) | n = 596 |
| head and neck squamous cell carcinoma (HNSC) | n = 598 |
| kidney chromophobe (KICH) | n = 112 |
| kidney renal clear cell carcinoma (KIRC) | n = 606 |
| kidney renal papillary cell carcinoma (KIRP) | n = 336 |
| lower grade glioma (LGG) | n = 551 |
| liver hepatocellular carcinoma (LIHC) | n = 450 |
| lung adenocarcinoma (LUAD) | n = 576 |
| lung squamous cell carcinoma (LUSC) | n = 554 |
| lymphoid neoplasm diffuse large B-cell lymphoma (DLBC) | n = 53 |
| mesothelioma (MESO) | n = 89 |
| ovarian serous cystadenocarcinoma (OV) | n = 582 |
| pancreatic adenocarcinoma (PAAD) | n = 207 |
| pheochromocytoma and paraganglioma (PCPG) | n = 189 |
| prostate adenocarcinoma (PRAD) | n = 573 |
| rectum adenocarcinoma (READ) | n = 170 |
| sarcoma (SARC) | n = 285 |
| skin cutaneous melanoma (SKCM) | n = 493 |
| stomach adenocarcinoma (STAD) | n = 450 |
| testicular germ cell tumors (TGCT) | n = 158 |
| thymoma (THYM) | n = 128 |
| thyroid carcinoma (THCA) | n = 588 |
| uterine carcinosarcoma (UCS) | n = 58 |
| uterine corpus endometrial carcinoma (UCEC) | n = 547 |
| uveal melanoma (UVM) | n = 81 |

**Fig. S1.** Protein-protein interaction of 38 inflammatory aging clock-related genes.

**
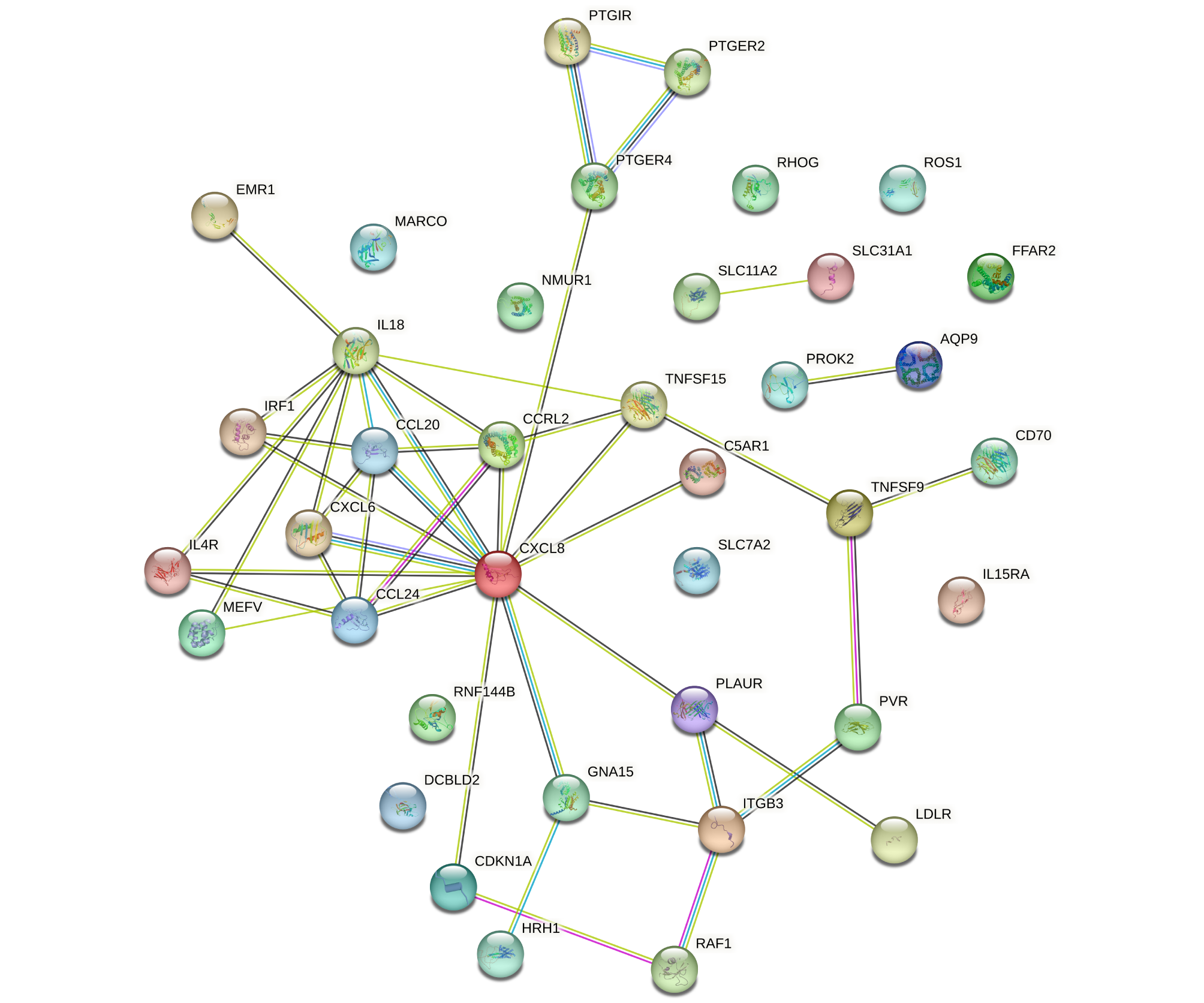
**
